# Accuracy of the PiCCO_2_-derived pulse contour cardiac index (CIpc): development and validation of a calibration index in two independent collectives

**DOI:** 10.1186/cc10829

**Published:** 2012-03-20

**Authors:** W Huber, J Koenig, B Saugel, T Schuster, R Schmid, S Mair

**Affiliations:** 1 Klinikum Rechts der Isar, Technical University of Munich, Germany

## Introduction

After calibration by thermodilution (TD), the PiCCO device is able to assess CO using pulse contour (PC) analysis. Despite an overall good correlation of CItd and CIpc in several studies, the manufacturer suggests recalibration by TD after 8 hours. A calibration index derived from PC parameters indicating a certain probability of a relevant bias and triggering the next calibration would be of great practical use. Therefore, it was the aim of our study to prospectively evaluate predictors of the bias CItd-CIpc exactly 1 hour, 2 hours, 4 hours, 6 hours and 8 hours after the last calibration.

## Methods

In 28 consecutive patients 56 datasets each including six TDs were recorded. In each triplicate TD measurement, CIpc was recorded immediately before recalibration by TD and compared to CItd. Results derived from this evaluation collective were validated in an independent second collective of 48 patients with 67 datasets. SPSS 19 software was used.

## Results

The sample was 19 males, nine females, age 60.2 ± 11.8 years; APACHE II score 23.3 ± 5.4. The 280 pairs of CIpc and CItd showed a significant correlation (*P *< 0.001; *r *= 0.907). There was no difference between CIpc versus CItd (4.15 ± 1.46 vs. 4.09 ± 1.41 l/minute*sqm; *P *= 0.265). Bland-Altman analysis demonstrated a mean bias of -0.061 ± 0.603 l/minute*sqm (lower and upper levels of agreement -1.24 and 1.12l/minute*sqm; percentage error of 28.7%). In univariate analyses, the bias CItd2-CIpc was not correlated to the interval to the last calibration (*P *= 0.705; *r *= -0.023), but it was correlated to CIpc immediately before recalibration (*r *= -0.275; *P *< 0.001) and to changes from CIpc versus the previous CItd1 (Delta-CIpc-CItd1; *r *= -0.504; *P *< 0.001). These findings were confirmed in the validation collective (*P *< 0.001). Multiple regression analysis demonstrated independent association of the bias to Delta-CIpc-CItd1. This association was best described by bias CItd2-CIpc = -0.014 - 0.372*x *+ 0.145*x*^2 ^- 1.260*x*^3 ^with *x = *Delta-CIpc-CItd1. This formula as a potential calibration index provided ROC AUCs of 0.882 and 0. 751 (*P *< 0.001) to predict a bias CItd2-CIpc >20% or <-20% in the evaluation collective. This formula was confirmed with ROC AUCs of 0.809 and 0.714 (*P *< 0,001) to predict a bias CItd2-CIpc >20% or <-20% in the independent validation collective.

## Conclusion

The difference CIpc-CItd1 is an independent predictor of the bias CItd2-CIpc. A calibration index was developed and validated. It could be a useful decision support to initiate the next TD.

